# The Etiological Spectrum of Febrile Encephalopathy in Adult Patients: A Cross-Sectional Study from a Developing Country

**DOI:** 10.1155/2018/3587014

**Published:** 2018-06-03

**Authors:** Elham Peidaee, Fereshte Sheybani, HamidReza Naderi, Nasrin Khosravi, Mehdi Jabbari Nooghabi

**Affiliations:** ^1^Faculty of Medicine, Mashhad University of Medical Sciences, Mashhad, Iran; ^2^Department of Infectious Diseases and Tropical Medicine, Faculty of Medicine, Mashhad University of Medical Sciences, Mashhad, Iran; ^3^Clinical Research Unit, Faculty of Medicine, Mashhad University of Medical Sciences, Mashhad, Iran; ^4^Center for Disease Control and Prevention, Mashhad University of Medical Sciences, Mashhad, Iran; ^5^Department of Statistics, Faculty of Mathematical Sciences, Ferdowsi University of Mashhad, Mashhad, Iran

## Abstract

The profile of febrile encephalopathy varies based on different demographic and geographical characteristics of the study population. This retrospective, cross-sectional study was conducted to evaluate the etiological spectrum of febrile encephalopathy in hospitalized adult patients. A total of 293 patients with the mean age of 49.7 ± 23 were evaluated of whom 77.1% presented with encephalopathy syndrome. The most common diagnosis in patients with clinical syndromes suggestive of central nervous system (CNS) infection was sepsis associated encephalopathy (SAE) (22.9%), followed by bacterial meningitis (14%) and neurotuberculosis (9.9%). The comparison between the elderly and young adults showed that, in the young adults, bacterial meningitis and neurotuberculosis, and in the elderly SAE, are among the most common causes of clinical syndromes suggestive of CNS infection including febrile encephalopathy in our region. Moreover, we illustrated an upward trend for the proportion of diagnosing CNS infections among those who underwent diagnostic LP, from 40.4% in 2011 to 70% in 2015, that could be indicative of an increasing threshold for performing LP at least in our center in recent years. Whether these changes have been associated with increasing the rate of diagnostic errors or not needs to be evaluated in future studies.

## 1. Introduction

The management of patients suffering from fever and altered mental status is one of the common concerns of physicians in emergency departments [1]. Considering the fact that confusion is a key sign of encephalopathy, this symptom accounts for around 2% of the patients in emergency departments [2]. The list of differential diagnosis of the clinical syndrome of febrile encephalopathy is long and timely differentiation between these disorders is very important because correct diagnosis and treatment have a significant impact on morbidity and mortality. This diagnostic challenge is especially important in dealing with patients with multiple chronic medical conditions [3]. The first challenge facing the emergency clinician is to define what is meant by an altered mental status or confusion and to ascertain why it led to a visit to the emergency department (ED) [2]. In such conditions, it is important to differentiate between infectious processes, autoimmune disorders, and encephalopathies. The latter refers to a noninflammatory diffuse cerebral dysfunction, mostly triggered by a number of metabolic or toxic conditions [4]. It is important to note that when cerebral dysfunction is accompanied by fever or sepsis syndrome, the possibility of an infectious process, especially pertaining to a central nervous system (CNS) infection, as an etiologic cause for an alteration in the mental status should be considered [3]. Since these potentially treatable infectious processes might be associated with significant morbidity and mortality, timely diagnosis and treatment are of great importance for saving a patient's life [5, 6].

The profile of* febrile encephalopathy varies on the basis* of* different* demographic and* geographical* characteristics of the study population [3]. It is important to determine the etiologic spectrum of febrile encephalopathy syndrome, with an emphasis on the CNS infection by focusing on epidemiology and age groups. The knowledge of these data is essential for protocol development at the regional level in order to appropriately manage patients. While such studies are commonly performed in the pediatric age group [7–11], there are few data for the adult population [12–15]. This study was conducted to determine the etiological spectrum of the febrile encephalopathy syndrome in hospitalized adult patients who underwent diagnostic lumbar puncture (LP) and to compare the clinical characteristics between elderly patients and young adults in Mashhad, Iran.

## 2. **Materials and Methods**

This retrospective cross-sectional study was carried out in a 1000-bed teaching hospital affiliated to Mashhad University of Medical Sciences, Mashhad, Iran. This hospital is one of the two main referral centers for adult patients with febrile encephalopathy in the northeast of Iran. In order to collect data, the Health Information System (HIS) was used to extract the list of all adult patients (≥15 years old) who had undergone diagnostic LP between 2011 and 2015. The patients were selected randomly based on the health record number. Almost all of the LP cases in our medical center are performed to rule in or rule out CNS infections (and with much less frequency to assess other differential diagnoses). Subsequently, the data were extracted and entered into the checklist. All clinical and paraclinical data were reviewed to verify the accuracy of the final diagnosis of patients (both syndromic and etiological diagnoses). The exclusion criteria included history of recent head trauma, history of recent neurosurgery within three months prior to admission, the presence of cerebrospinal fluid (CSF) shunt, and verifying the diagnosis of ischemic or hemorrhagic stroke as the etiology of cerebral dysfunction.

### 2.1. Definitions

*Elderly* refers to an adult older than 65 years of age.

Encephalopathy is defined as a clinical state of altered mental status, manifested as confusion, disorientation, behavioral changes, or other cognitive impairments, with or without inflammation of the brain tissue [16].

Encephalitis is defined as an inflammation of the brain parenchyma associated with neurologic dysfunction usually resulting from a direct infection of the brain parenchyma, postinfectious processes, or noninfectious conditions. In the absence of pathological evidence of cerebral inflammation, an inflammatory response in the CSF or the presence of abnormalities in the brain parenchyma following neuroimaging is used as alternative markers of brain inflammation [16].

*Level of consciousness *is determined on the basis of Glasgow coma scale (GCS). Impaired level of consciousness is defined as Glasgow coma scale < 15.

*Sepsis associated encephalopathy* (SAE) is defined as a diffuse brain dysfunction secondary to infection elsewhere in the body without overt CNS infection [17].

*Presumptive Diagnosis*. Because of limitations such as low sensitivity of assays for the detection of pathogens responsible for CNS infections, we reported the final diagnosis in two categories: presumptive and definitive diagnoses. Presumptive diagnosis is defined as the most probable etiologic diagnosis based on the clinical and paraclinical findings despite negative microbiological confirmatory tests. For instance, in patients with acute meningitis without positive gram stain and culture results that CSF parameters were in favor of bacterial meningitis and complete resolution of the illness achieved during or after an antimicrobial therapy, the diagnosis was presumptively reported as bacterial meningitis.

### 2.2. Statistical Analysis and Sample Size

Data were described by using descriptive statistical methods, including frequency tables, statistical charts, central tendency, and dispersion indices. The research objectives were analyzed by using the chi-squared test, Fisher's exact test, and Student's *t*-test, as well as ANOVA or nonparametric tests formulated by Mann–Whitney and Kruskal–Wallis. The descriptive methods of the Shapiro–Wilk test and Lilliforse's test were applied to evaluate the normal distribution of the quantitative data.

### 2.3. Ethical Considerations

The Ethical Committee of Mashhad University of Medical Sciences approved the present study assigned with the code number of IR.MUMS.fm.REC.1394.578.

## 3. Results

In total, out of the 590 patients who had undergone diagnostic LP during the study period, 300 cases were selected randomly, and seven of them were excluded from the study based on the exclusion criteria. Finally, 293 patients were enrolled in the study. The mean age of the patients was 49.7 ± 23 (15–95), including 178 (60.8%) males and 115 (39.2%) females with a male to female ratio of 1.54. The mean lag time from symptom onset to admission was 9.77 ± 7.64 (1–90).

The underlying disorders in decreasing orders of frequency were hypertension/ischemic heart disease (*n* = 77, 26.3%), diabetes mellitus (*n* = 45, 15.4%), history of cerebrovascular accident (CVA) (*n* = 31, 10.6%), bedridden status (*n* = 29, 9.9%), psychiatric disorders (*n* = 21, 7.2%), dementia/Alzheimer's disease, (*n* = 18, 6.1%), receiving immunosuppressive medications (*n* = 11, 3.8%), chronic pulmonary disease (*n* = 10, 3.4%), chronic kidney disease/renal failure (*n* = 8, 2.7%), Parkinson's disease (*n* = 7, 2.4%), hematological malignancies/oncological disorders (*n* = 6, 2%), rheumatologic disorders (*n* = 5, 1.7%), and other chronic medical conditions (*n* = 33, 11.2%). A history of previous hospitalization within three months was found in 52 (17.7%) patients and residency in long term care facilities in eight (7.2%) patients.

The percentage frequency distribution of the main finding within the first hours of admission that led to the decision of performing LP consisted of altered mental status (*n* = 192, 65.8%), headache without impairment in mental status (*n* = 78, 26.7%), signs of meningeal irritation (*n* = 7, 2.4%), seizure (*n* = 5, 1.7%), and others including transient impairment of consciousness and oscillatory levels of consciousness (*n* = 10, 3.3%). Overall, there were signs of meningeal irritation in 157 (53.6%) patients and the results of the assessment were reported as negative in 102 (34.8%) cases. The presence or absence of meningeal irritation could not be interpreted because of chronic underlying conditions in 16 (5.5%) cases. In 18 (6.1%) patients, nothing was mentioned about meningeal irritation examination in the health records.

**Figure 1:**
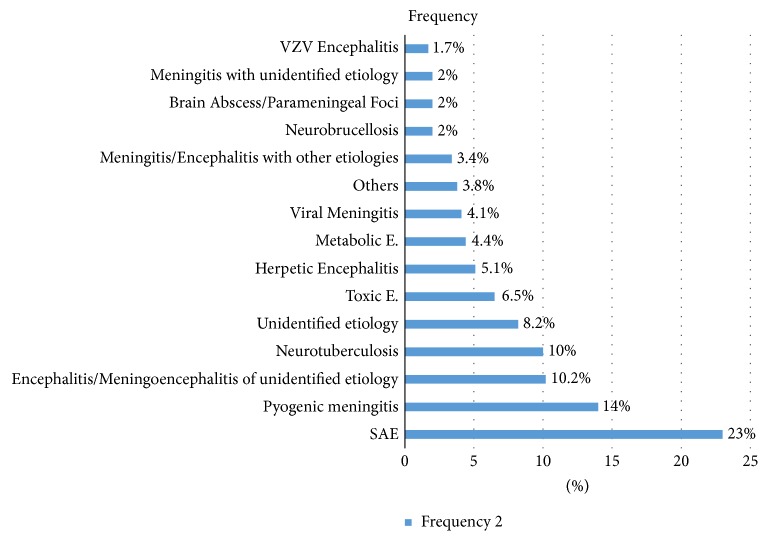
Final diagnosis of clinical syndromes suggestive of CNS infection. Metabolic E: Metabolic Encephalopathy; Toxic E: Toxic Encephalopathy; SAE:* Sepsis Associated Encephalopathy;* VZV:* Varicella Zoster Virus*.

The final diagnoses in decreasing orders of frequency were SAE (*n* = 67, 22.9%), bacterial meningitis (*n* = 41, 14%), encephalitis/meningoencephalitis of undetermined etiology (*n* = 30, 10.2%), neurotuberculosis (*n* = 29, 9.9%), unidentified etiology (*n* = 24, 8.2%), toxic encephalopathy (*n* = 19, 6.5%), herpes simplex encephalitis (*n* = 15, 5.1%), metabolic encephalopathy (*n* = 13, 4.4%), viral meningitis (*n* = 12, 4.1%), meningitis, or encephalitis with other etiologies including cryptococcal meningitis, autoimmune encephalitis, drug-induced meningitis, neuro-Behçet's disease and CNS complications of lupus erythematosus (*n* = 11, 3.4%), neurobrucellosis (*n* = 6, 2%), parameningeal infections/brain abscess (*n* = 6, 2%), meningitis of undetermined etiology (*n* = 6, 2%), Varicella Zoster Virus (VZV) encephalitis (*n* = 5, 1.7%), and others (*n* = 11, 3.8%) (see Figure 1). The other causes included leptomeningeal carcinomatosis, postictal state, and exacerbation of preexisting psychiatric disorders. Two patient had both bacterial meningitis and brain abscesses simultaneously.

The underlying infections in the SAE cases in decreasing orders of frequency were pleuropulmonary infections (*n* = 36, 53.7%), sepsis with unknown source (*n* = 16, 23.9%), urinary tract infection (*n* = 3, 4.5%), bacteremia (*n* = 3, 4.5%), and others including intra-abdominal infection, soft tissue infection, infective endocarditis, and septic arthritis (*n* = 9, 3%). In 158 (53.9%) patients, the final diagnosis was a CNS infection. The information on the percentage frequency distribution and etiological spectrum of patients with CNS infection is provided in Table 1.

The frequency of pathogens isolated from patients with microbiologically documented CNS infection was as follows:* S. pneumoniae* (*n* = 14, 9.8%),* Mycobacterium tuberculosis* (*n* = 12, 22.6%), herpes simplex virus (*n* = 8, 15.1%),* Brucella* species (*n* = 6, 11.3%),* Staphylococcus aureus*,* Pseudomonas* species, and* Acinetobacter* species (*n* = 1, 1.9%, each), and other cases (*n* = 4, 7.5%). Six (11.3%) cases of CNS infection were gram stain positive, culture negative clinical specimens. In 103 (65.1%) cases with CNS infections, the etiology of the disease was not microbiologically documented.

Of bacterial meningitis, 24 (57.5%) cases were microbiologically documented. The etiological diagnosis of bacterial meningitis was verified in 10 (43.5%) cases with both gram stain and culture, in one (4.3%) case with culture alone, and in 12 (52.2%) cases with gram stain alone. Only five (12.2%) patients with bacterial meningitis had positive blood culture results. The most common causative agent in the microbiologically documented cases of bacterial meningitis was* S. pneumoniae* (60.8%).

In the present study, 226 (77.1%) patients had encephalopathy syndrome at the time of diagnosis. The percentage frequency of final diagnosis in this group of patients was as follows: SAE (*n* = 61, 27%), bacterial meningitis (*n* = 27, 11.9%), encephalitis or meningoencephalitis of undetermined etiology (*n* = 27, 11.9%), neurotuberculosis (*n* = 20, 8.8%), toxic encephalopathy (*n* = 19, 8.4%), unidentified etiology (*n* = 16, 7.1%), herpes simplex encephalitis (*n* = 15, 6.6%), metabolic encephalopathy (*n* = 13, 5.8%), parameningeal infections/brain abscesses (*n* = 5, 4.2%), meningitis/encephalitis with other etiologies (*n* = 9, 4%), neurobrucellosis (*n* = 2, 2.9%), Varicella Zoster Virus encephalitis (*n* = 5, 2.5%), viral meningitis (*n* = 3, 1.3%), and others (*n* = 6, 2.7%).

**Figure 2:**
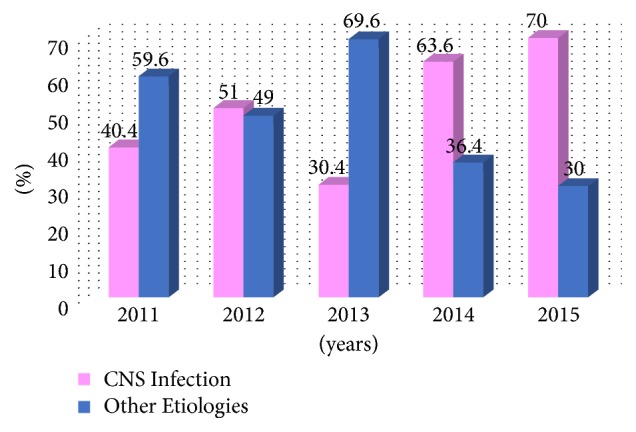
The proportion of patients with CNS infections to all cases with febrile encephalopathy.

The proportion of CNS infections to all the cases with febrile encephalopathy who had undergone LP based on the year of hospitalization was 19 (40.4%) of 47 in 2011, followed by 26 (51%) of 51 in 2012, 17 (30.4%) of 56 in 2013, 14 (63.6%) of 22 in 2014, and 35 (70%) of 50 in 2015 (Figure 2).

**Figure 3:**
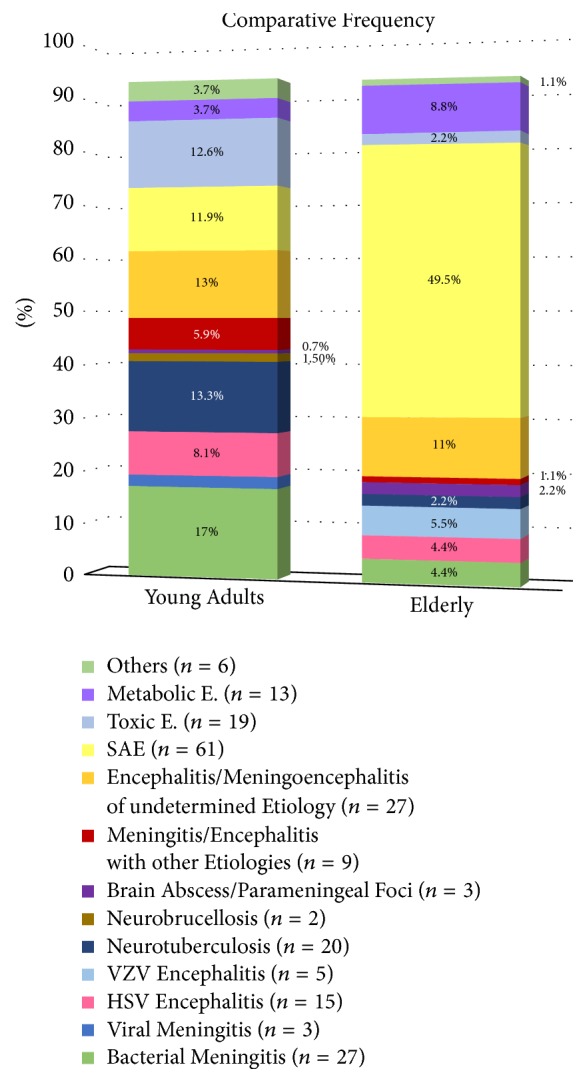
The comparison of frequency distribution of the final diagnosis in patients with febrile encephalopathy syndrome between the elderly (≥65 years) and young adults (<65 years). HSV:* Herpes Simplex Virus*; VZV:* Varicella Zoster Virus*;* SAE*: Sepsis Associated Encephalopathy;* E*.: Encephalopathy.

The frequency distribution of the final diagnosis in patients with febrile encephalopathy syndrome based on age groups is listed in Figure 3.

Finally, 48 (16.4%) patients died, 227 (77.7%) survived and were discharged from hospital, and the clinical outcome remained unknown for 17 (5.8%) patients due to transfer to another hospital or because of the patient leaving the hospital against medical advices.

The comparison of the characteristics between patients with SAE and those with CNS infection is shown in Table 2. We also compared the characteristics of patients with SAE and the subgroup of CNS infection with encephalopathy syndrome (Table 2).

Fourteen percent of patients with CNS infection died, while the mortality rate was 21.2% in cases without CNS infection (*P* value = 0.12).

## 4. Discussion

According to this study, the most common diagnosis in patients with clinical syndromes suggested CNS infection was SAE (23%), followed by bacterial meningitis, neurotuberculosis, toxic encephalopathy, and herpes simplex encephalitis. In general, a few points were important in this study: first, a high proportion of neurotuberculosis in patients with febrile encephalopathy syndrome requiring hospitalization; second, bacterial meningitis and neurotuberculosis being the most common etiologies of febrile encephalopathy syndrome, as well as CNS infections in young adults; third, a high proportion of SAE in the elderly age group with febrile encephalopathy; and fourth, a high proportion of meningoencephalitis of undetermined etiology. No presumptive or definitive etiology was verified in about 12% of the patients with meningitis or meningoencephalitis syndromes; and fifth, the relatively high proportion of CNS infections with gram stain positive, culture negative clinical specimens or totally negative microbiological results as compared to the microbiologically documented cases. In more than half of the patients with SAE, the underlying infection had a pleuropulmonary focus. The comparison of the two groups of SAE and subgroup of CNS infection with encephalopathy syndrome revealed that the SAE patients were often older adults in bedridden status with a history of multiple underlying conditions such as dementia, recent CVA, or the presence of diabetes mellitus with lower level of GCS. However, the frequency of positive meningeal signs was significantly higher in those with CNS infection.

The etiological spectrum of febrile encephalopathy varied across different geographic regions, as well as on the basis of the age range of the participants and the study population [3]. Several studies have investigated the etiological spectrum of febrile encephalopathy and the appropriate threshold for urgent diagnostic evaluation to either verify or rule out CNS infection in the pediatric age group [18, 19]. However, there are few similar studies in the case of adults. A literature review found several studies that reported CNS infections to be the most common causes of changes in mental status in children with nontraumatic coma [7–9]; however, this finding has not been observed in all studies [20]. Moreover, it is unclear whether this is also true in adult patients with similar clinical presentation or not. In a large study performed in China [21], infectious syndromes, including CNS infections, accounted for only 13.1% of all 1934 adult patients with undifferentiated altered mental status at a single center tertiary care academic emergency department. Several other studies on the etiological spectrum of febrile encephalopathy in adults have been published from India. In two of them that provided information about Indian patients with an average age of 30 to 40 years, bacterial meningitis, viral encephalitis and SAE, followed by tuberculous meningoencephalitis, cerebral malaria, leptospirosis, and brain abscesses, were reported as the most common causes of febrile encephalopathy [12, 15]. In another study from India in which one-third of the participants were elderly, meningitis was responsible for more than half of the cases with acute encephalitis syndrome, followed by metabolic encephalopathy, alcoholic encephalopathy, cerebral malaria, brain abscesses, and SAE [16].

Despite limited information about the etiological spectrum of febrile encephalopathy in adults, many studies across the world have investigated the etiological spectrum of specific syndromes of CNS infections, including encephalitis syndrome in different populations [22–26]. A recently published large retrospective multinational study (*n* = 2583) has provided information on the etiological spectrum of community acquired CNS infections from 37 referral centers in 20 countries [27]. The most frequent infecting pathogens reported in this study were* Streptococcus pneumoniae* and* Mycobacterium tuberculosis *[27], which are the same as in our study. The results of numerous studies that investigated the etiological spectrum of encephalitis syndrome differed according to the populations studied, the geographic regions, and diagnostic methodologies, as well as the “definition of case” used for encephalitis syndrome. In most of these studies, the most common causes of encephalitis were reported to be herpes viruses, especially HSV and VZV [22–24]. However, M. tuberculosis was reported as the most prevalent cause in a study in England [26] and as the second leading cause of encephalitis in a study in France [25].

As a developing country, Iran is also faced with the problem of limited information about the profile of febrile encephalopathy, as well as the microbial spectrum of CNS infections. However, observational studies, including case series and case reports or small cross-sectional studies, have illustrated CNS infections as an important challenge to physicians in Iran. There are few retrospective reviews of the etiological spectrum of CNS infections over a period of one or several years [28], or the final diagnosis of hospitalized patients with possible CNS infection from Iran [14]. The only report on the incidence of meningitis in Iran (1999–2005) has estimated the incidence rate of 1 to 12.8 per 100,000 populations in Tehran in different age groups [29]. In a systematic review (2016) of acute bacterial meningitis in Iran,* S. pneumoniae* was reported as the most prevalent causative pathogen of bacterial meningitis [30]. Despite common bacterial and viral agents responsible for CNS infections, other endemic and rare pathogens, including rabies virus [31],* M. tuberculosis* [32, 33],* Brucella* species [34, 35],* Bacillus anthracis* [36],* Borrelia recurrentis* [37],* Plasmodium* species [38],* Echinococcus* species [39],* Naegleria fowleri* [40],* Cryptococcus neoformans* [41], Prions [42], and many others have been reported in studies on CNS infections from Iran but mostly described as case reports or small case series. In our study, the diagnosis of only around one-third of the patients with CNS infections was documented microbiologically. Similar to previous studies, the most prevalent pathogen of bacterial meningitis, as well as CNS infections, was* S. pneumoniae*. Despite the reported yields of 70–85% for CSF culture, as well as 50–90% for blood culture in bacterial meningitis [43, 44], the yields in our study were only around 25% and 12%, respectively. There could be several possible explanations for the relatively low rate of microbiological documentation that was observed in our study and some previous studies from Iran [45, 46], including the higher proportion of patients who received antimicrobials before presentation, delay in performing diagnostic tests including LP, improper collection of* clinical specimen* and transport in the environment to the laboratory, inadequately accurate culture-based microbiological techniques [47], and the limited use of molecular diagnostic tests such as PCR, except for certain pathogens such as herpes simplex virus in our healthcare centers.

Lumbar puncture (LP) is one of the most valuable diagnostic measures for verifying or ruling out CNS infection. Although the number of definite indications for LP has been reduced with the onset of new diagnostic methods, especially neuroimaging techniques, urgent LP to diagnose CNS infections is still indicated [48]. It is unclear as to which threshold is appropriate for performing LP in patients with clinical syndromes suggestive of CNS infection in different age groups in order to minimize diagnostic errors. In other words, it is uncertain as to how many LPs should be performed to diagnose one case of CNS infection. While some studies have examined the threshold of performing LP in children with fever and seizure or neonates with febrile syndromes [18, 19], there are no similar studies regarding the appropriate threshold in adults. Our study demonstrated an upward trend, from around 40% in 2011 to around 70% in 2015, for the proportion of the diagnosis of CNS infections among those who underwent diagnostic LP that could be indicative of an lowering threshold for performing LP, at least in our center, in recent years. Whether these changes have been accompanied by increasing rate of missed or delayed diagnosis or better screening and reduced medical costs is a topic that needs further investigation, including autopsy-based studies. Although our study demonstrated several factors that were more evident in patients with SAE compared to those with CNS infection and encephalopathy, we cannot recommend the non-performance of LP in older adults having a bedridden status with a history of multiple underlying conditions such as dementia, recent CVA, or diabetes mellitus that is present with febrile encephalopathy. However, it can be suggested to not perform LP as the first diagnostic procedure in the first minutes to hours of presentation of a patient with febrile encephalopathy with the mentioned characteristics and delay it until other more common etiologies of encephalopathy have been excluded. In other words, the appropriate threshold for performing LP in the first hours of evaluation of this group of patients might be higher in comparison to other patients with syndromes suggestive of CNS infection.

This study has some inherent strength such as the reporting of the diseases in both syndromic and etiologic diagnoses, as well as presumptive and definitive diagnoses. However, it had several limitations as well. First, this study was a retrospective analysis of patients. Second, the investigation was carried out in a single academic center, thereby reducing its generalizability to the general population. Third, the outcome of the patients who had sought discharge against medical advice remained unknown.

## 5. Conclusions

The knowledge about the etiological spectrum of febrile encephalopathy across different geographic regions as well as for different age groups is a necessity for protocol development at the regional level. The current study demonstrated high proportion of SAE among elderly patients with febrile encephalopathy as well as high proportion of neurotuberculosis and bacterial meningitis among adult patients with CNS infections. It also reported a high proportion of meningoencephalitis of undetermined etiology and relatively low rate of microbiological documentation in CNS infections in our region. Moreover, we proposed a possible decrease in the threshold for performing LP in recent years. Whether these changes have been accompanied by an increased rate of missed or delayed diagnosis or not needs to be evaluated in future studies.

## Figures and Tables

**Table 1:** Frequency distribution and etiological spectrum of patients with CNS infection.

	Definitive diagnosis	Presumptive diagnosis	Undetermined etiology	Total
	*n* (%)	*n* (%)	*n* (%)	*n*
Bacterial meningitis	23 (56.1)	18 (43.9)	-* *-	*41*
Viral meningitis	0 (0)	12 (100)	-* *-	*12*
Herpetic encephalitis	8 (53.3)	7 (46.7)	-* *-	*15*
VZV encephalitis	0 (0)	5 (100)	-* *-	*5*
CNS tuberculosis	12 (41.3)	17 (58.6)	-* *-	*29*
Neurobrucellosis^*∗*^	6 (100)	0 (0)	-* *-	*6*
Parameningeal infection/brain abscess	1 (25)	3 (75)	-* *-	*4*
Meningitis/encephalitis of other etiologies	3 (30)	7 (70)	-* *-	*10*
Meningitis of undetermined etiology	-* *-	-* *-	6 (100)	*6*
Encephalitis/meningoencephalitis of undetermined etiology	-* *-	-* *-	30 (100)	*30*

Total	53 (33.9)	68 (43.6)	35 (22.4)	*158*

^*∗*^The diagnosis of neurobrucellosis was made by serology and/or culture; VZV: *Varicella Zoster Virus*; CNS: *central nervous system*.

**Table 2:** The comparison of the characteristics between patients with CNS infection and SAE.

	SAE	CNS infection	*P* value	CNS infection with encephalopathy syndrome	*P* value
Age, years (mean)	68.26	42.57	**<0.001**	48.12	**<0.001**
Underlying Conditions					
Bedridden status	22 (33.8)	2 (1.3)	**<0.001**	2 (1.9)	**<0.001**
Dementia	11 (16.9)	4 (2.6)	**<0.001**	4 (3.7)	**0.004**
Diabetes (DM)	16 (24.6)	14 (9.1)	**0.002**	13 (12)	**0.032**
HTN/IHD	26 (40)	31 (20.1)	**0.002**	30 (27.8)	0.096
CVA	15 (23.1)	7 (4.5)	**<0.001**	7 (6.5)	**0.002**
Psychiatric disorder	5 (7.7)	7 (4.5)	0.263	4 (3.7)	0.253
Duration of illness, days (mean)	5.67	8.24	**<0.001**	8.11	0.900
GCS (mean)	11.95			12.5	**0.045**
Meningeal Signs	26 (38.8)	100 (63.7)	**0.002**	66 (59.5)	**0.035**
Seizure	7 (10.4)	22 (14.1)	0.287	22 (20)	0.085
Leukocytosis (WBC ≥ 12000)	28 (43.1)	51 (32.5)	0.134	39 (35.1)	0.295
ESR (mean)	41.95	30.34	0.290	34.89	0.157
Hyponatremia (Na < 135)	11 (16.4)	32 (20.5)	0.477	23 (20.9)	0.462
In Hospital Mortality	17 (25.4)	20 (12.7)	**0.028**	19 (18.6)	0.198

HTN/IHD: hypertension/ischemic heart disease; CVA: cerebrovascular accident; GCS: *Glasgow Coma Scale*; FNDs: focal neurologic deficit; WBC: white blood cell; ESR: erythrocyte sedimentation rate.
